# The Flexural Strength and the Effect of the Autoclave Sterilization of Polypropylene/Natural Rubber Blended Materials

**DOI:** 10.3390/dj12110361

**Published:** 2024-11-13

**Authors:** Paphavarin Rangsantham, Thitaporn Nonthiphalang, Panjaporn Wongwitthayakool, Chakrit Sirisinha, Nantawan Krajangta, Panupat Phumpatrakom

**Affiliations:** 1 Division of Endodontics, Faculty of Dentistry, Thammasat University, Pathumthani 12121, Thailand; paphavarin.ran@dome.tu.ac.th; 2 Division of Restorative Dentistry, Faculty of Dentistry, Thammasat University, Pathumthani 12121, Thailand; thitaporn.non@dome.tu.ac.th (T.N.); knantawa@tu.ac.th (N.K.); 3 Division of Oral Biology, Faculty of Dentistry, Thammasat University, Pathumthani 12121, Thailand; panja_w@tu.ac.th; 4 Rubber Technology Research Center (RTEC), Faculty of Science, Mahidol University, Nakhon Pathom 73170, Thailand; chakrit.sir@mahidol.ac.th

**Keywords:** flexural strength, natural rubber, polypropylene, rubber dam clamp

## Abstract

**Background:** Rubber dam clamps are used extensively in dentistry, especially during root canal treatment. However, existing rubber dam clamps have several drawbacks, including discomfort and potential damage to vital tissue in the oral cavity. To address these existing issues, a new rubber dam clamp should be developed. The aim of this study was to identify the optimum ratios of polypropylene and natural rubber (PP/NR) for a customized rubber dam clamp in dentistry. This study was focused on the flexural strength of PP/NR in various ratios. Moreover, the impact of autoclave sterilization was also considered. **Methods:** Six proportions of PP/NR blends (100/0, 90/10, 80/20, 70/30, 60/40 and 50/50) were prepared and assessed for flexural strength using a three-point bending test. After this test, the PP/NR blends with 100/0, 90/10 and 80/20 ratios were selected and underwent autoclave sterilization for 1, 5 and 10 cycles. Eventually, the flexural strength testing was repeated and investigated. An analysis of variance and Tukey’s test were used to evaluate the flexural strength of various PP/NR blends before autoclave sterilization at *p* < 0.05. An analysis of variance and Dunnett’s T3 test were used to evaluate the flexural strength of selected PP/NR blends before and after autoclave sterilization at *p* < 0.05. **Results:** The results revealed that the flexural strength of PP/NR blended materials showed a statistically significant difference in every group of this study. The autoclave sterilization test revealed that the flexural strength of the PP/NR 90/10 and 80/20 ratios was significantly increased after sterilization for 1, 5 and 10 cycles. In addition, the PP/NR 90/10 ratio was also comparable to the 100/0 ratio. The lower NR content in PP/NR blends resulted in significantly higher flexural strength, and autoclave sterilization had an effect on this property. **Conclusions:** This study suggested that the PP/NR blend with a 90/10 ratio might be considered as an alternative material for developing rubber dam clamps.

## 1. Introduction

The rubber dam isolation technique offers many advantages in root canal treatment and restorative procedures. It prevents the contamination of the operative field by saliva, blood and gingival crevicular fluid, protects oral soft tissue from exposure to root canal irrigating solutions and reduces the risk of the accidental ingestion of dental instruments. The rubber dam is often held in place using a rubber dam clamp that fits the greatest convexity of each tooth [1,2]. Metal is the material for a conventional rubber dam clamp. The advantages of a metal clamp are rigidity, stability and the ability to be sterilized. However, metal clamps can cause patient discomfort, potential damage to gingival tissue, tooth structure and dental restorations [1,3,4]. Moreover, the metal clamp can be distorted over time, leading to improper seating on the tooth and potential dislodgement [5]. Recently, non-metal or polymer rubber dam clamps have been developed to avoid dental and gingival tissue damage [6]. They result in less pressure on the tooth surface, making the patient more comfortable. Softclamp™ (Kerr, Bioggio, Switzerland) is a commercially available non-metal rubber dam clamp constructed from polyether ether ketone (PEEK), a high-performance thermoplastic recognized for its toughness, rigidity and strength [7,8]. However, this clamp has some drawbacks, including a short lifespan and high cost. To overcome the limitations of existing rubber dam clamps, a new rubber dam clamp should be further developed. The desired rubber dam clamp should provide stable retention on the tooth, reduce damage to dental and gingival tissue, offer flexibility and adaptability to the tooth, provide durability for clinical use and be autoclavable.

Polypropylene (PP) is a widely used thermoplastic polymer due to its unique properties such as a high melting temperature, low density and substantial chemical and heat resistance. PP is notably resistant to cracking under bending stress, making it the preferred material for applications requiring bending tolerance [9,10]. Natural rubber (NR) is also classified as a polymer and plays a role in the local economy. It is extensively used in dental materials such as elastic ligatures, elastic chains and rubber dam sheets due to its high flexibility and elasticity [11]. NR provides good tensile and tear strength at a reasonable cost. Additionally, it can be compounded to achieve a variety of properties [12,13]. The mixing of various kinds of polymers is an effective way to create blends with better characteristics than their individual components. NR blended with thermoplastics like PP has been extensively researched to improve properties including the strength, toughness and hardness [14,15]. The blending ratio can be adjusted to produce materials with varying mechanical properties, ranging from soft elastomers to semi-rigid plastics [10]. So, the desired properties of the rubber dam clamp might be achieved through the blending of PP and NR.

Flexural strength is a parameter that represents a material’s ability to resist bending deformation under an applied load [16]. Flexural strength testing is required since some parts of the rubber dam clamp require bending tolerance.

Autoclave sterilization is a highly effective method for sterilizing and disinfecting medical equipment through the use of heat, pressure and time [17]. A previous study investigated the effect of steam sterilization on metal rubber dam clamps and found no detrimental effect on their mechanical properties [5]. However, research on polymer rubber dam clamps has not been conducted, and the autoclave capability of PP/NR blends remains in question and should be elucidated.

Therefore, this study aimed to compare the flexural strength of PP/NR blends in various ratios. After identifying suitable ratios, the effects of autoclave sterilization were evaluated for the further development of customized rubber dam clamps. The following null hypotheses were investigated: (1) the composition of PP/NR blends shows no influence on the flexural strength and (2) the autoclave sterilization shows no influence on the flexural strength of the selected suitable PP/NR blends.

## 2. Materials and Methods

Polypropylene and natural rubber were used to prepare thermoplastic polyolefins (TPOs), a simple blend in which the rubber phase is not vulcanized. A commercially available polypropylene (EL-Pro™, SCG Performance Chemical, Bangkok, Thailand) with a melting temperature of 169 °C was used as the major blend component. The natural rubber, STR5L grade (RAOT Nakhonsithammarat, Thailand), was used as received.

### 2.1. Specimen Preparation

The materials were mixed in a laboratory-size internal mixer (Haake Rheocord 90 Fisons, Berlin, Germany) with a mixing chamber volume of 80 cm^3^. The blend composition ratios of PP/NR were 100/0, 90/10, 80/20, 70/30, 60/40 and 50/50% by weight.

The components were melt-blended at 175 °C. The process began by melting the PP pellets in the mixing chamber for 3 min. Once the PP had melted, small lumps of NR were added, and blending continued for an additional 7 min to ensure a uniform mixture. The mixing curve was used to monitor the homogeneity of the melt. This procedure was repeated for different PP/NR ratios.

The specimens were prepared via compression molding using a hydraulic hot press (CT compression machine, Chareon Tut, Samut Prakan, Thailand) at 175 °C for 10 min, then cooling them down to room temperature under pressure for 5 min. The specimen was removed from the mold and examined for voids or chipped edges. Any defective specimens were discarded.

The sample size for each experimental group was 8 specimens in accordance with the ASTM D790 standard for flexural property testing, which requires at least 5 specimens per group [16]. In addition, G*Power 3.1.9.6 (University of Düsseldorf, Düsseldorf, Germany) [18] was used to calculate the sample size, and the effect size (Cohen’s f) was estimated using the results of our pilot study. G*Power indicated that the sample size used in this test had a power of >0.95 in a one-way ANOVA (α = 0.05).

**Figure 1:**
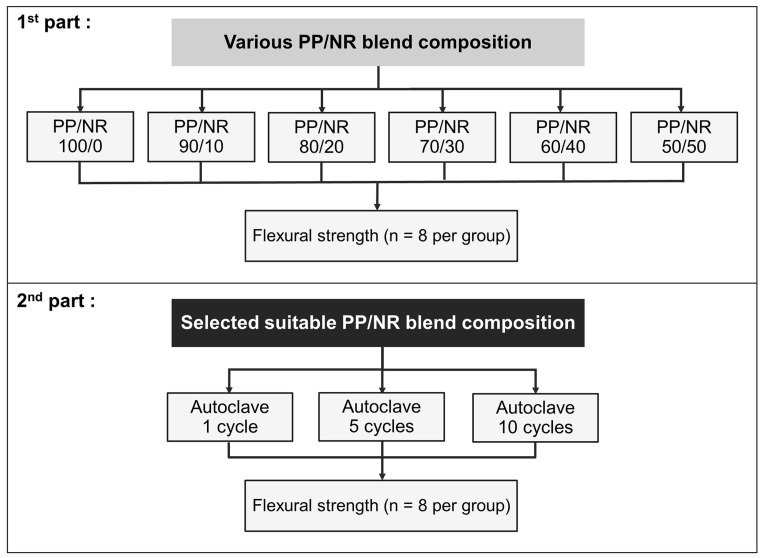
The flow chart for the study design.

Firstly, this study examined the flexural strength (n = 8) of various PP/NR blends to find suitable ratios. Secondly, the selected PP/NR blends were tested for the effect of autoclave sterilization on the flexural strength (n = 8) after 1, 5 and 10 autoclave sterilization cycles. The flow chart for the study design is presented in Figure 1.

### 2.2. Flexural Strength Testing

**Figure 2:**
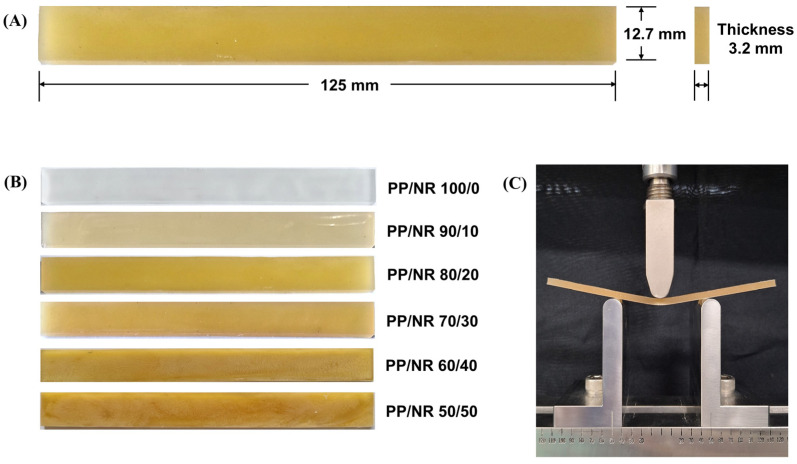
(**A**) Flexural strength specimen dimensions; (**B**) examples of test specimens; and (**C**) placing the specimen on the two supporting units (50 mm span length) and applying force until failure.

The flexural strength test specimens were prepared with dimensions of 12.7 × 125 × 3.2 mm^3^ according to the ASTM D790 standard (Figure 2A,B) [18]. The specimens’ dimensions were verified using a caliper (Mitutoyo, Kawasaki, Japan).

Flexural strength testing was carried out using a universal testing machine (AGSX, Shimadzu, Tokyo, Japan) with a three-point bending test in accordance with the ASTM D790 standard [16]. Each specimen was placed on two supporting units separated by 50 mm and tested with a 500 N load cell at a crosshead speed of 1.3 mm/min until failure occurred (Figure 2C). Then, the maximum force was recorded. The flexural strength (σ; MPa) was calculated using Equation (1).

(1) $$\sigma =\frac{3FL}{2bh^{2}}$$

where *F* = the applied load at fracture or failure (N).

*L* = the distance between the supports or span length (mm).

*b* = width of the specimens (mm).

*h* = thickness of the specimens (mm).

### 2.3. Autoclave Sterilization

The selected PP/NR blends from the first part of the study were used to evaluate the effects of autoclave sterilization. After 1, 5 and 10 cycles, the flexural strength was measured. The changes observed in the flexural strength value following autoclave sterilization were used to assess their resistance to the sterilization process.

For the autoclave sterilization process, the specimens were placed in an autoclave (MML-Bester, Med tech LTD., Bangkok, Thailand) and subjected to steam at 121 °C and 15 psi of pressure. Each sterilization cycle consisted of a 20 min hot cycle followed by a 30 min drying period within the autoclave. After each cycle, the specimens were left to dry overnight before undergoing the next sterilization cycle.

### 2.4. The Data’s Statistical Assessment

Data analysis was conducted using SPSS software (SPSS version 26.0, SPSS Inc., Chicago, IL, USA). The Shapiro–Wilk test was used to assess the normality of the data. A one-way analysis of variance (ANOVA), followed by Tukey’s post hoc test, was used to compare the flexural strength of various PP/NR blends prior to autoclave sterilization. The influence of autoclave sterilization (before and after 1, 5 and 10 cycles) on the flexural strength of selected PP/NR blends was evaluated using a one-way ANOVA, followed by Dunnett’s T3 test for multiple comparisons. The statistical significance was set at *p* < 0.05.

## 3. Results

**Figure 3:**
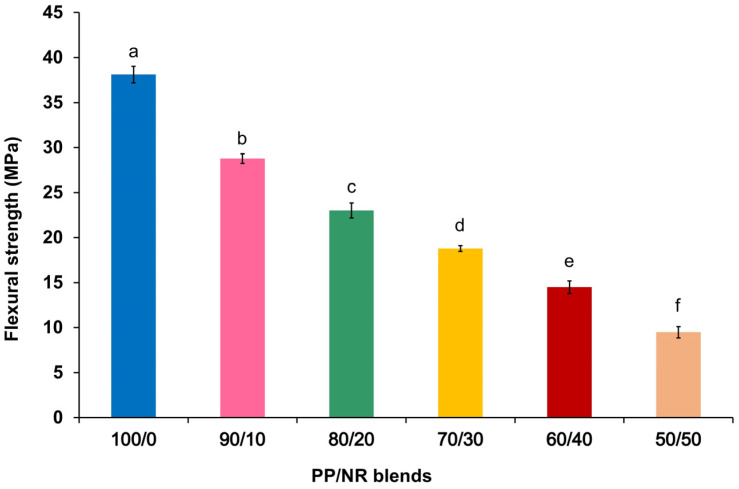
Flexural strength of PP/NR blends before autoclave sterilization. The same lowercase letters indicate no significant difference between groups.

For the flexural strength analysis, all test groups met the assumption of normality. The mean and standard deviation (SD) of the flexural strength before autoclave sterilization are presented in Figure 3. Significant differences were found among the different PP/NR blends (*p* < 0.001). The flexural strength of blends with low NR content was significantly higher than those with high NR content (*p* < 0.001).

**Figure 4:**
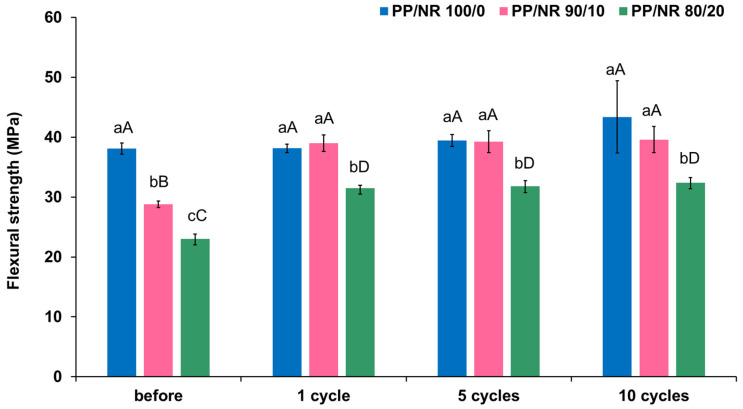
Flexural strength of selected PP/NR blends before and after autoclave sterilization cycles. The same lowercase letters indicate no significant difference within groups of the same autoclave cycle. Similarly, the same uppercase letters denote no significant difference between each PP/NR ratio across different autoclave cycles.

The mean and standard deviation (SD) of the flexural strength of three selected PP/NR blends before and after autoclave sterilization are presented in Figure 4. PP/NR blends with 90/10 and 80/20 blend compositions showed a significant increase after autoclave sterilization (*p* < 0.001). Meanwhile, following one autoclave sterilization cycle, the 90/10 PP/NR blend demonstrated flexural strength comparable to pure PP (*p* = 0.980). After 5 and 10 autoclave sterilization cycles, the flexural strength of the 90/10 PP/NR blend remained comparable to pure PP (*p* = 1.000 and 0.964, respectively).

## 4. Discussion

Rubber dam clamps play a crucial role in dentistry, particularly during root canal treatment. However, conventional metal rubber dam clamps have several drawbacks, including discomfort and potential damage to vital tissue [1,3,4]. This study assesses the impact of combining polypropylene (PP) and natural rubber (NR) on the flexural strength, as well as the influence of autoclave sterilization. As the various ratios of PP/NR blends showed an influence on flexural strength, the first null hypothesis was rejected. The second null hypothesis was partially rejected since differences in flexural strength were observed for 90/10 and 80/20 PP/NR blends before and after autoclave sterilization.

To date, there is no internationally accepted standard for the production and quality evaluation of non-metal rubber dam clamps [19]. A previous report by Jacoby in 1988 stated that typical rubber dam clamps for holding the dam against canines or bicuspids require a flexural force of approximately 10–20 pounds (23.96–47.94 MPa) to separate the clamp wings far enough apart to fit over the tooth. This force is also sufficient to hold the dam against the tooth when the clamp returns to its unseparated position. Larger clamps used for molars would require a greater separation force [20]. Consequently, this study utilizes the flexural strength value as stated previously to serve as a reference for comparison.

The results revealed that the composition of PP/NR blends affects flexural strength. Moreover, the selected PP/NR blends demonstrated significantly higher flexural strength after autoclave sterilization. A high flexural strength in PP/NR blends is necessary to ensure the materials can resist bending deformation under applied mechanical force. It was observed that the highest flexural strength in PP/NR blends was found in the 100/0 blend (pure PP), followed by the 90/10, 80/20, 70/30, 60/40 and 50/50 blends, respectively. The addition of NR to PP resulted in a significant reduction in flexural strength across all PP/NR blends due to the inherently soft nature of NR when added to the more rigid PP phase [9], as well as the immiscible blend of PP and NR, which was caused mainly by poor interfacial adhesion, with the increasing rubber content causing a reduction in the rigidity and an increase in the elastomeric nature of the blend [21].

The mean flexural strengths of the 100/0, 90/10 and 80/20 PP/NR blends were close to that of typical rubber dam clamps. Therefore, these three ratios were selected to assess the durability after autoclave sterilization. The flexural strength post-autoclave sterilization of the 90/10 and 80/20 PP/NR blends significantly increased compared to their pre-autoclave states. In addition, the 90/10 PP/NR blend was comparable to the pure PP. This could be due to the recrystallization of the PP phase (annealing effect) during sterilization. Post-thermal annealing, also known as heat treatment, is commonly used to reduce weak bonds and enhance overall mechanical integrity. Thermal annealing works by allowing polymer chains to diffuse across strand boundaries at elevated temperatures, hence improving strand–strand interfacial performance. Annealing-induced crystallization is commonly observed in semi-crystalline polymers, including PP, and has been correlated with improved mechanical performance [22,23]. As a result, the findings indicated sterilization resistance.

Flexural strength testing was conducted at a crosshead speed of 1.3 mm/min in accordance with the ASTM D790 standard, which is the standard test method for unreinforced and reinforced plastics and is generally applicable to rigid and semirigid materials, including PP/NR blends [16]. This current study was also consistent with another dental-related study that used a rate of 1.3 mm/min to investigate the flexural properties of thermoplastic polymer materials for the 3D printing of dental restorations [24].

The autoclave sterilization test in this study was limited to ten cycles based on research indicating that most plastics can withstand one–five cycles of steam sterilization at 121 °C [17]. Our results were consistent with another study conducted by Fischer and Howells in 2021, which investigated the re-usability of autoclaved 3D-printed PP compared to glass-filled polypropylene composite (GFPP), and found that the higher temperature of 134 °C was excessive, resulting in the melting of PP and GFPP within two–three autoclave cycles. PP and GFPP, on the other hand, showed minor changes (<1%) in mass and volume following one, four, seven and ten autoclave cycles at 121 °C [25]. Thus, the current study set the temperature for autoclave sterilization at 121 °C for 10 cycles.

The current study can only state that the flexural strength of selected PP/NR blends (100/0, 90/10 and 80/20) both pre- and post-autoclave sterilization was higher than the flexural strength reported in 1988 [20], and there has been no additional published research to date.

A limitation of this study was the lack of a standard comparator for non-metallic rubber dam clamps. The study was initially designed to use the Softclamp™ for comparison, but the remelting process to mold the test specimen was not feasible due to its high melting temperature (343 °C) [8]. As a result, this study relied only on the reference value as previously mentioned. In addition, autoclave sterilization was limited to 10 cycles; thus, future research should use more autoclave cycles to assess the long-term mechanical properties of this material. Future studies should also consider other properties such as dynamic mechanical properties that demonstrate the material’s viscoelastic behavior, the stress distribution of the 3D-printed PP/NR clamp retained on the tooth, and clinical relevance tests such as clinician and patient satisfaction assessments.

## 5. Conclusions

Based on the findings of this study, the following conclusions were drawn:

1. The lower NR content in PP/NR blends resulted in significantly higher flexural strength.
2. Autoclave sterilization had an effect on the flexural strength of PP/NR blends. After autoclave sterilization, the 90/10 ratio showed flexural strength comparable to pure PP. However, the 90/10 and 80/20 ratios exhibited flexural strength with an acceptable range for customized rubber dam clamps. This implied that the 90/10 PP/NR blend is suitable as a candidate for developing rubber dam clamps.

## Data Availability

Data are contained within the article.
